# Supplementary material for: Classification of sleep apnea syndrome using the spectrograms of EEG signals and YOLOv8 deep learning model
Source: PeerJ Comput Sci. 2025 Feb 21;11:e2718. doi: 10.7717/peerj-cs.2718 (PMC11888935; doi:10.7717/peerj-cs.2718)
Supplement: Supplemental Information 1 — There are codes for 3 deep learning classifier models. [file peerj-cs-11-2718-s001.docx]

% segmentation and spectrogram

data = load('your data here');

signal = data.your data name;

file_name='your data';

Fs = 128;

segment_length = 30;

num_segments = floor(length(signal) / (segment_length * Fs));

window = hamming(segment_length * Fs);

noverlap = floor(0.5 * length(window));

nfft = 2^nextpow2(length(window));

save_directory = 'your directory here';

for i = 1:num_segments

start_index = (i - 1) * segment_length * Fs + 1;

end_index = min(i * segment_length * Fs, length(signal));

segment = signal(start_index:end_index);

[S, F, T] = spectrogram(segment, window, noverlap, nfft, Fs);

h = figure('Visible', 'off');

imagesc(T, F, 10*log10(abs(S)));

axis xy;

xlabel('Time (s)');

ylabel('Frequency (Hz)');

title(sprintf('Segment %d Spectrogram', i));

colorbar;

file_name = sprintf('%s_spectrogram_%d.png',file_name,i);

full_file_dir = fullfile(save_directory, file_name);

saveas(h, full_file_dir);

end

close(h);

% train/val/test separation

import os

import random

import shutil

dataset_dir = "/your directory here"

folders= ["your spectrogram folders here"]

train_ratio = 0.7

val_ratio = 0.15

test_ratio = 0.15

for folder in folders:

    folder_dir = os.path.join(dataset_dir, folder)

    for k in ["train", "val", "test"]:

        os.makedirs(os.path.join(folder_dir, k), exist_ok=True)

   features = os.listdir(folder_dir)

    for feature in features:

        feature_dir = os.path.join(folder_dir, feature)

        pictures = os.listdir(feature_dir)

        random.shuffle(pictures)

        train_number = int(len(pictures) * train_ratio)

        val_number = int(len(pictures) * val_ratio)

% ResNet64 Classifier

!pip list | grep -e fastai -e torch -e torchvision

!pip install roboflow

from roboflow import Roboflow

rf = Roboflow(api_key="YOUR API KEY HERE")

project = rf.workspace("your workspace name here").project("your Project name here")

dataset = project.version(2).download("folder")

import os

labels = os.listdir("Your directory here")

print("No. of labels: {}".format(len(labels)))

print("-----------------")

for label in labels:

    print("{}, {} files".format(label, len(os.listdir("your directory here"+label))))

import numpy as np

import matplotlib.pyplot as plt

from PIL import Image

fig, ax = plt.subplots(nrows=2, ncols=2)

fig.tight_layout()

cnt = 0

for row in ax:

    for col in row:

        image_name = np.random.choice(os.listdir("your directory here"+ labels[cnt]))

        im = Image.open("your directory here{}/{}".format(labels[cnt],image_name))

        col.imshow(im)

        col.set_title(labels[cnt])

        col.axis('off')

        cnt += 1

plt.show()

from fastai.vision import *

from fastai.metrics import accuracy

import torchvision.transforms as transforms

from torchvision import datasets

from torch.utils.data import DataLoader

path = "your data directory here"

mean = [0.485, 0.456, 0.406]

std = [0.229, 0.224, 0.225]

normalize = transforms.Normalize(mean=mean, std=std)

transform = transforms.Compose([

    transforms.Resize(size),

    transforms.ToTensor(),

    normalize,

])

dataset = datasets.ImageFolder(root=path, transform=transform)

batch_size = bs

data_loader = DataLoader(dataset, batch_size=batch_size, shuffle=True, num_workers=4)

for batch in data_loader:

    images, labels = batch

from fastai.vision.all import *

data = DataBlock(blocks=(ImageBlock, CategoryBlock),

                 get_items=get_image_files,

                 splitter=RandomSplitter(valid_pct=0.2, seed=42),

                 get_y=parent_label,

                 item_tfms=Resize(460),

                 batch_tfms=[*aug_transforms(size=224), Normalize.from_stats(*imagenet_stats)])

dls = data.dataloaders(path)

learner = cnn_learner(dls, models.resnet34, metrics=[accuracy, Precision(average='macro'), Recall(average='macro'), F1Score(average='macro')])

learner.fine_tune(20)

interp = ClassificationInterpretation.from_learner(learner)

interp.plot_confusion_matrix()

import torch

import torch.nn as nn

from torchsummary import summary

from torchvision.models import resnet50  # ya da diğer ResNet modellerini seçebilirsiniz

class ResNet64(nn.Module):

    def __init__(self):

        super(ResNet64, self).__init__()

        self.resnet = resnet50()

    def forward(self, x):

        return self.resnet(x)

model = ResNet64()

print(model)

input_channels, height, width = 3, 224, 224

summary(model, input_size=(input_channels, height, width))

% YOLOv5 Classifier

!git clone https://github.com/ultralytics/yolov5  # clone

%cd yolov5

%pip install -qr requirements.txt  # install

import torch

import utils

display = utils.notebook_init()  # checks

from utils.downloads import attempt_download

p5 = ['n', 's', 'm', 'l', 'x']  # P5 models

cls = [f'{x}-cls' for x in p5]  # classification models

for x in cls:

    attempt_download(f'weights/yolov5{x}.pt')

#Infer using classify/predict.py

!python classify/predict.py --weights ./weigths/yolov5s-cls.pt --source bananas.jpg

# Ensure we're in the right directory to download our custom dataset

import os

os.makedirs("../datasets/", exist_ok=True)

%cd ../datasets/

# REPLACE the below with your exported code snippet from above

!pip install roboflow

from roboflow import Roboflow

rf = Roboflow(api_key="YOUR API KEY")

project = rf.workspace("yolov5-classification").project("banana-ripeness-classification")

dataset = project.version(1).download("folder")

#Save the dataset name to the environment so we can use it in a system call later

dataset_name = dataset.location.split(os.sep)[-1]

os.environ["DATASET_NAME"] = dataset_name

%cd ../yolov5

!python classify/train.py --model yolov5s-cls.pt --data $DATASET_NAME --epochs 20 --img 128 --pretrained weights/yolov5s-cls.pt

!python classify/val.py --weights runs/train-cls/exp/weights/best.pt --data ../datasets/$DATASET_NAME

#Get the path of an image from the test or validation set

if os.path.exists(os.path.join(dataset.location, "test")):

  split_path = os.path.join(dataset.location, "test")

else:

  os.path.join(dataset.location, "valid")

example_class = os.listdir(split_path)[0]

example_image_name = os.listdir(os.path.join(split_path, example_class))[0]

example_image_path = os.path.join(split_path, example_class, example_image_name)

os.environ["TEST_IMAGE_PATH"] = example_image_path

print(f"Inferring on an example of the class '{example_class}'")

#Infer

!python classify/predict.py --weights runs/train-cls/exp/weights/best.pt --source $TEST_IMAGE_PATH

#Directory infer

os.environ["TEST_CLASS_PATH"] = test_class_path = os.path.join(*os.environ["TEST_IMAGE_PATH"].split(os.sep)[:-1])

print(f"Infering on all images from the directory {os.environ['TEST_CLASS_PATH']}")

!python classify/predict.py --weights runs/train-cls/exp/weights/best.pt --source /$TEST_CLASS_PATH/

% YOLOv8 Classifier

import os

HOME = os.getcwd()

print(HOME)

# Pip install method (recommended)

!pip install ultralytics

from IPython import display

display.clear_output()

import ultralytics

ultralytics.checks()

from ultralytics import YOLO

%cd {HOME}

%cd {HOME}

Image(filename='runs/classify/predict/dog.jpeg', height=600)

!yolo task=classify mode=predict model=yolov8n-cls.pt conf=0.25 source='https://media.roboflow.com/notebooks/examples/dog.jpeg'

from IPython.display import display, Image

model = YOLO(f'{HOME}/yolov8n-cls.pt')

results = model.predict(source='https://media.roboflow.com/notebooks/examples/dog.jpeg', conf=0.25)

!mkdir {HOME}/datasets

%cd {HOME}/datasets

!pip install roboflow --quiet

from roboflow import Roboflow

rf = Roboflow(api_key="YOUR_API_KEY")

project = rf.workspace("roboflow-universe-projects").project("banana-ripeness-classification")

dataset = project.version(4).download("folder")

%cd {HOME}

!yolo task=classify mode=train model=yolov8n-cls.pt data={dataset.location} epochs=20 imgsz=128

!ls -la {HOME}/runs/classify/train/

!cat {HOME}/runs/classify/train/results.csv | head -10

%cd {HOME}

!yolo task=classify mode=val model={HOME}/runs/classify/train/weights/best.pt data={dataset.location}

%cd {HOME}

!yolo task=classify mode=predict model={HOME}/runs/classify/train/weights/best.pt conf=0.25 source={dataset.location}/test/overripe

import glob

from IPython.display import Image, display

for image_path in glob.glob(f'{HOME}/runs/classify/predict/*.jpg')[:3]:

      display(Image(filename=image_path, width=600))

      print("\n")

project.version(dataset.version).deploy(model_type="yolov8-cls", model_path=f"{HOME}/runs/classify/train/")
